# Adult-Onset ANCA-Associated Vasculitis in SAVI: Extension of the Phenotypic Spectrum, Case Report and Review of the Literature

**DOI:** 10.3389/fimmu.2020.575219

**Published:** 2020-09-29

**Authors:** Frederik Staels, Albrecht Betrains, Peter Doubel, Mathijs Willemsen, Vincent Cleemput, Steven Vanderschueren, Anniek Corveleyn, Isabelle Meyts, Ben Sprangers, Yanick J. Crow, Stephanie Humblet-Baron, Adrian Liston, Rik Schrijvers

**Affiliations:** ^1^Department of Microbiology, Immunology and Transplantation, Allergy and Clinical Immunology Research Group, KU Leuven, Leuven, Belgium; ^2^Department of Microbiology, Immunology and Transplantation, Immunogenetics Research Group, KU Leuven, Leuven, Belgium; ^3^Department of Microbiology, Immunology and Transplantation, Laboratory for Clinical Infectious and Inflammatory Disease, KU Leuven, Leuven, Belgium; ^4^Department of Nephrology, AZ Groeninge, Kortrijk, Belgium; ^5^VIB-KU Leuven Center for Brain and Disease Research, Leuven, Belgium; ^6^Department of Pathology, University Hospitals KU Leuven, Leuven, Belgium; ^7^Laboratory for Molecular Diagnosis, Center for Human Genetics, KU Leuven, Leuven, Belgium; ^8^Laboratory of Inborn Errors of Immunity, Department of Microbiology, Immunology and Transplantation, KU Leuven, Leuven, Belgium; ^9^Department of Microbiology, Immunology and Transplantation, Molecular Immunology, KU Leuven, Leuven, Belgium; ^10^Centre for Genomic Medicine, MRC Institute of Genetics and Molecular Medicine, The University of Edinburgh, Edinburgh, United Kingdom; ^11^Laboratory of Neurogenetics and Neuroinflammation, Université de Paris, Paris, France; ^12^Laboratory of Lymphocyte Signalling and Development, Babraham Institute, Cambridge, United Kingdom

**Keywords:** SAVI, vasculopathy, glomerulonephritis, primary immunodeficiency, interferonopathy

## Abstract

STING-associated vasculopathy with onset in infancy (SAVI) is an autosomal dominant disorder due to gain-of-function mutations in *STING1*, also known as *TMEM173*, encoding for STING. It was reported as a vasculopathy of infancy. However, since its description a wider spectrum of associated manifestations and disease-onset has been observed. We report a kindred with a heterozygous STING mutation (p.V155M) in which the 19-year-old proband suffered from isolated adult-onset ANCA-associated vasculitis. His father suffered from childhood-onset pulmonary fibrosis and renal failure attributed to ANCA-associated vasculitis, and died at the age of 30 years due to respiratory failure. In addition, an overview of the phenotypic spectrum of SAVI is provided highlighting (a) a high phenotypic variability with in some cases isolated manifestations, (b) the potential of adult-onset disease, and (c) a novel manifestation with ANCA-associated vasculitis.

## Introduction

STING-associated vasculopathy with onset in infancy (SAVI) was initially reported as a vasculopathy of infancy. However, since its initial description, a wider spectrum of associated manifestations and disease-onset has been observed. We report a kindred with isolated adult-onset ANCA-associated vasculitis, extending the phenotypic spectrum. In addition, we provide a comprehensive overview of the phenotypic spectrum of SAVI by reviewing the other 54 cases reported in literature. Our findings highlight (a) a high phenotypic variability with in some cases isolated manifestations, (b) the potential of adult-onset disease, and (c) a novel manifestation with ANCA-associated vasculitis.

## Case Report

**FIGURE 1:**
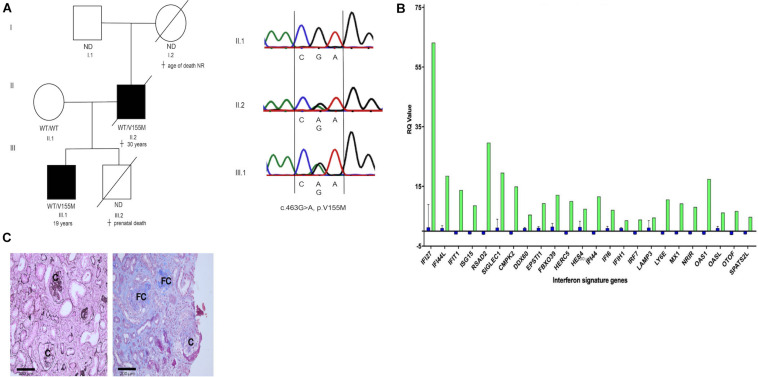
**(A)** Pedigree: c.463G > A, p.V155M mutation in *STING1* was confirmed by Sanger in proband III.1 and II.2. **(B)** Relative quantification (RQ) of interferon stimulated genes on qPCR of whole blood RNA of III.1 (green) and 27 controls (blue). **(C)** Renal biopsy of II.2 (left panel) and III.1 (right panel) showing extra and intracapillary with glomerulonephritis cellular **(C)** and fibrocellular (FC) crescents.

A 19-year-old Caucasian male (Figure 1A, III.1, and Supplementary Document E1 and Supplementary Table E1, available in the online repository) presented with exertional dyspnea and fatigue. Eleven months prior to the current presentation he reported an episode of hemoptysis, but left without further investigation. Initial blood results demonstrated a profound microcytic (MCV 76.8 fL, reference 82–98 fL) anemia (Hb 6.3 g/dL, reference 13–16 g/dL) with iron deficiency (transferrin saturation 6.6%, 20–50%) and mildly elevated C-reactive protein (23.1 mg/L, reference 0–5 mg/L). Gastro- and colonoscopy did not reveal gastrointestinal bleeding. He received red blood cell transfusion and was discharged with iron and vitamin supplements. One week later, he presented with a painful, red eye and decreased vision. A diagnosis of iritis was made. Further work-up revealed the presence of myeloperoxidase (MPO) specific antineutrophil cytoplasmic antibodies (ANCA, titer 1/640) with proteinuria (1.76 g/24 h, reference <0.14 g/24 h), pyuria (25/μL, reference <10/μL) and hematuria (185/μL, reference <10/μL, 88% dysmorphic) on urinalysis. Subsequent renal biopsy identified a pauci-immune focal crescentic and necrotizing glomerulonephritis with mesangial C3 deposition (Figure 1C). His father (Figure 1A, II.2) had suffered from childhood-onset pulmonary fibrosis and renal failure attributed to ANCA-associated vasculitis with progressive pauci-immune intra- and extra-capillary glomerulonephritis, dying at the age of 30 years due to respiratory failure (Figure 1C). The patient’s brother died *in utero* with renal vein thrombosis as the only notable finding on autopsy. Given the family history, whole-exome sequencing was performed, revealing a known pathogenic c.463G > A (p.V155M) mutation in *STING1* (1). Sanger sequencing confirmed the presence of this variant in the proband and his father but not in his healthy mother (Figure 1A). Interferon (IFN) stimulated genes (ISG) expression, determined by qPCR on patient’s whole blood RNA was increased compared to controls (Figure 1B). Hence, a diagnosis of autosomal dominant STING-associated vasculopathy with onset in infancy (SAVI) (1) was made, presenting in our proband as adult-onset isolated renal and ocular ANCA-associated vasculitis.

## Review of the Literature

**FIGURE 2:**
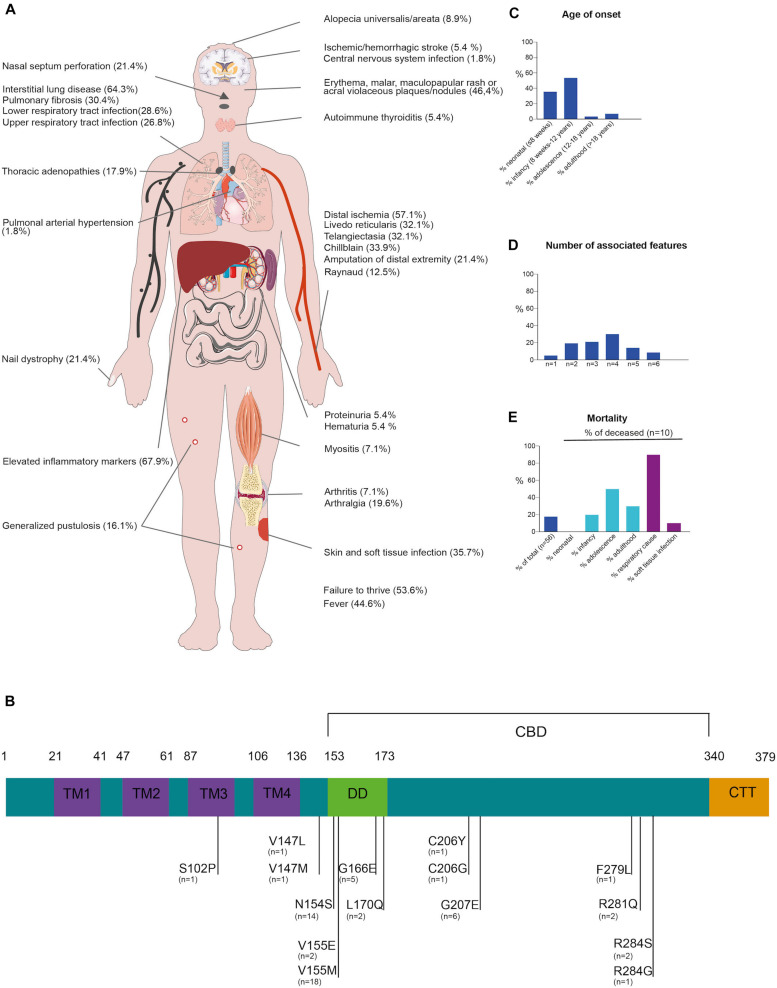
Clinical and genetic synopsis of 56 SAVI patients. **(A)** Schematic representation of potential clinical presentations **(B)** Schematic representation of the STING protein, consisting of 4 transmembrane domains (TM1-4, blue), dimerization domain (DD, green), cGAMP binding domain (CBD, black bar) and C-terminal tail domain (CTT, orange). Gain-of-function mutations are indicated in black with number of cases reported underneath **(C)** Age of onset, *n* = 52 reported **(D)** Number of associated features, *n* = 56 reported **(E)** Mortality in SAVI patients, *n* = 56 reported.

To further outline the phenotypic spectrum, the clinical manifestations, genetics and treatment modalities in 56 genetically confirmed SAVI patients (including our kindred) was reviewed (Figure 2 and Supplementary Figure E1 and Supplementary Table E1, available in the online repository).

Disease-onset usually manifested early in life (35.7% in neonatal life and up to 92.9% presenting before adulthood). Adult-onset SAVI had only been described in two other patients, a male with a long-standing history of arthralgia and recurrent elevated inflammatory parameters and a 20-year-old female with interstitial lung disease (ILD), progressing to lung fibrosis requiring lung transplantation (2, 3). SAVI has a high mortality rate, with 18% of reported patients deceased at the time of publication, mostly due to respiratory complications and before adulthood (Figure 2) (1–3).

Across the 56 surveyed patients, vasculopathy resulting from vasculitis and endothelial cell death is a hallmark of SAVI mostly affecting the skin, lungs and central nervous system (1, 4). Clinically this commonly manifested as chilblains (33.9%), telangiectasia (32.1%), livedo reticularis (32.1%) and Raynaud phenomena (12.5%). Sometimes, more severe manifestations occurred such as acral ischemia necessitating amputations (21.4%) or ischemic/hemorrhagic stroke (5.4%) (1, 4). Cutaneous manifestations were common in SAVI, mostly with erythematous, malar, maculopapular rashes, and acral violaceous plaques (46.4%) with or without concomitant nail dystrophy (21.4%). Skin biopsy (performed in 37.5%) often reveals (peri-)vascular inflammation involving small arteries and capillaries with variable cellular infiltrates, C3 deposition and intravascular thrombi (1, 5). In one patient, granulomatous nodular dermatitis and secondary fibrosis were observed.

Pulmonary involvement was also common (69.6%), most frequently manifesting as ILD (64.3%) with infiltrative interstitial opacification in the lung periphery on CT-scan. In a substantial number of cases (30.4%), ILD progressed to pulmonary fibrosis. Other manifestations included intrathoracic lymphadenopathy (17.9%). Lung biopsies performed in 28.6% predominantly identified lymphocytic infiltrates surrounding alveoli and bronchioles (1–3).

STING-associated vasculopathy with onset in infancy patients are susceptible to soft tissue (35.7%) and respiratory tract (55.4%) infections, which can be related to their underlying vascular or pulmonary disease, as most patients had severe digital ischemia or underlying ILD or fibrosis. However, SAVI itself may carry an infectious susceptibility as a considerable percentage of patients had lymphopenia, leukopenia or impaired lymphocyte proliferation tests (1, 2, 6). Immunophenotyping in our proband also showed CD4 + T-cell, NK-cell lymphopenia and impaired T-cell proliferation (Supplementary Table E1). Arthralgia, myalgia and arthritis, mainly affecting the small joints, were noted in a 21.4% of patients. Renal manifestations are rare (7.1%). One patient of African-American ethnicity, presenting with skin vasculopathy and ILD, developed generalized edema due to nephrotic range proteinuria at the age of 14 months (4). Renal biopsy showed focal segmental glomerulosclerosis (4). Of note, this patient also carried two *APOL1* risk variants which are associated with this kidney disease. Another patient had mild renal involvement with microscopic hematuria and hypertension requiring treatment (7). However, no renal biopsy was performed. In our kindred, a pauci-immune intra- and extra-capillary glomerulonephritis with proteinuria and hematuria was observed.

Blood analysis across SAVI patients typically showed elevated C-reactive protein and sedimentation rate (67.9%), indicating systemic inflammation. Auto-immune serology was often determined and up to 62% of SAVI patients had positive autoantibodies (Supplementary Figure E1) mostly anti-nuclear antibody, followed by ANCA, anti-cardiolipin antibody, lupus anticoagulant, and anti-phospholipid antibody. These autoantibodies confound the diagnosis of SAVI, as some patients are initially classified as systemic lupus or ANCA-vasculitis (8) patients based on their presentation and serology.

Treatment with corticosteroids, disease-modifying anti-rheumatic drugs, anti-TNF, anti-CD20 and intravenous immunoglobulins in SAVI patients has had limited or no effect. Based on the pathophysiology, treatment with JAK-inhibitors was evaluated in a number of patients with, albeit with varying success (see Supplementary Document E1, available in the online repository). In the patient described here, treatment with rituximab, followed by glucocorticoids and azathioprine resulted in a remission of his ocular features and partial remission of renal disease up to 16 months of follow-up (Supplementary Table E1).

## Conclusion

STING-associated vasculopathy with onset in infancy was initially identified in patients with early onset skin vasculopathy, ILD and prominent systemic features caused by *de novo* or familial gain-of-function mutations in STING. More recent reports have indicated a wider phenotypic spectrum including infectious, auto-immune, and even renal manifestations as in our case, with considerable variability between and within kindreds despite the presence of identical mutations. This clinical heterogeneity remains to be explained.

## Data Availability Statement

The raw data supporting the conclusions of this article will be made available by the authors, without undue reservation.

## Ethics Statement

The studies involving human participants were reviewed and approved by Ethical Committee of the University Hospitals of Leuven. The patients/participants provided their written informed consent to participate in this study. Written informed consent was obtained from the individual(s) for the publication of any potentially identifiable images or data included in this article.

## Author Contributions

FS, PD, BS, and RS initiated the work. FS, AB, MW, SV, IM, BS, YJC, SH-B, AL, and RS wrote the manuscript. PD provided the clinical care of the index patient. VC, YJC, and AC provided technical and/or diagnostic support. All authors contributed to the article and approved the submitted version.

## Conflict of Interest

The authors declare that the research was conducted in the absence of any commercial or financial relationships that could be construed as a potential conflict of interest.
