# Supplementary material for: Adult-Onset ANCA-Associated Vasculitis in SAVI: Extension of the Phenotypic Spectrum, Case Report and Review of the Literature
Source: Front Immunol. 2020 Sep 29;11:575219. doi: 10.3389/fimmu.2020.575219 (PMC7550674; doi:10.3389/fimmu.2020.575219)
Supplement: Supplementary file 1 [file Data_Sheet_1.docx]

**DOCUMENT E1 :** Supplementary information containing:

- Materials and methods
- Supplementary information on SAVI and reported SAVI patients
- Supplementary Table E1
- Supplementary Figure E1

*Materials and methods*

For DNA extraction Purelink DNA Genomic DNA Mini kit (ThermoFisher Scientific) was used according to the manufacturers’ instructions. DNA from the proband and mother was isolated from whole blood and for the proband additional Sanger sequencing confirmed on a hair sample (to exclude the presence of a revertant). For the deceased father a formalin-fixed paraffin-embedded renal tissue block stored at the department of Pathology was used to extract DNA. Sanger sequencing was done by Eurofins Genomics (Belgium). Whole exome sequencing was performed using SureSelect Human All Exon V7 (Agilent) for exome capture (Macrogen®, the Netherlands).

For NanoString ISG analysis, total RNA was similarly extracted from whole blood with a PAXgene (PreAnalytix) RNA isolation kit. Analysis of 24 genes and 3 housekeeping genes was conducted using the NanoString customer designed CodeSets according to the manufacturer’s recommendations (NanoString Technologies, Seattle, WA). Agilent Tapestation was used to assess the quality of the RNA. 100ng of total RNA was loaded for each sample. Data were processed with nSolver software (NanoString Technologies Seattle, WA). The data was normalized relative to the internal positive and negative calibrators, the 3 reference probes and the control samples. The median of the 24 probes for each of 27 healthy control samples was calculated. The mean NanoString score of the 27 healthy controls +2SD of the mean was calculated. Scores above this value (>2.724) were designated as positive.

For data collection, a comprehensive literature search was done in two electronic databases (MEDLINE and EMBASE) with following terms: TMEM173, STING, STING associated vasculopathy of infancy, SAVI and type I interferonopathy and relevant references within the retrieved articles were evaluated.

Ethics statement**:** research on patient cells was approved by the Ethical Committee of the University Hospitals of Leuven (approval number S58466). The patient and controls in this study provided informed consent.

*Supplementary information on SAVI and reported SAVI patients*

**Genetics**

The majority of *STING1* gain-of-function mutations reside in or near the dimerization domain (DD) or the cyclic dinucleotide binding domain (CBD) of the STING protein (Figure 2B). They are either inherited in an autosomal dominant manner or arise as *de novo* mutations. All functionally validated mutations reside in the coding region and result in constitutively active STING, either by stabilizing the STING homodimer (V155M, N154S, V147L and G166E) [1][E1-2], by inducing a conformational change affecting post-translational events and/or protein-protein interactions that spontaneously enable STING trafficking and signaling without the requirement for association with agonists such as cGAMP (R281Q, R284S/G, G207E, C206Y/G, S102P-F279L) [E3-7] or by another yet undetermined mechanism (V147M/L, V155E-L170Q).

**Genotype-phenotype relationship**

The genotype and phenotype seem to be poorly correlated. The first described mutations V155M, N154S and V147L have been reported with neonatal onset vasculitis, systemic inflammation and ILD [1]. Later, identical mutations were identified in other kindreds with a less severe disease course, absence or the single presence of either skin, vascular or pulmonary manifestations and in some individuals a later onset phenotype [2, 4][E8]. Inheritance seems to play a role, as *de novo* *STING1* mutations were more likely to have an early onset and severe phenotype, where familial inherited mutations had later onset and milder clinical symptoms. In later reports, other mutations were reported in the CBD or DD domain with a variable phenotype of STING-associated autoinflammation [E2, E3]. Remarkably some of these mutations were associated with an isolated phenotype such as familial chilblain lupus (G166E) or skin vasculopathy associated with autoimmunity manifesting as alopecia and hypothyroidism (G207E)[E2, E3]. All of these reported mutations led to excessive IFN-β production, but the molecular mechanism underlying the variable genotype-phenotype relation were unclear.

Current evidence points towards intrinsic genetic and environmental factors as modifiers of disease [6][E2, E9, E10]. In a recent report, Keskitalo et al. identified a kindred with autosomal inheritance of a G207E mutation and variable penetrance in skin vasculopathy, autoimmune phenomena and infection susceptibility [E2]. Common single nucleotide polymorphisms (SNPs) in IFIH1 (rs1990760; A946T, rs3747517; H843R) and STING (rs1131769; R232) were hypothesized to at least partially account for this heterogeneous presentation, as patients with the R232 variant in STING and/or both IFIH1 risk variants (T946, R843) had a more severe phenotype. This correlated with higher IFN regulated mRNA levels, a higher IFN signature and modest upregulation of the JAK-STAT pathway components in G207E patients with both IFIH1 risk variants (T946, R843) compared to a patient with only one risk variant (R843). The R232 variant in STING led to its enhanced activation, resulting in an amplified IFN-β response *in vitro*. Of note, the patient with all three variants (STING R232; IFIH1 T946 and R843) had the most severe phenotype, suggesting an additive effect of these polymorphisms to the G207E mutant. The putative enhanced effect of the R232 variant on clinical severity was also suspected by Malle et al. when they studied 9 SAVI patients with variable presentation and severity of ILD [E11]. Patients who were homozygous for the R232 variant had a more severe ILD course than heterozygous patients and one patient without ILD was homozygous for H232. Transfection of HEK293T cells with the R232 haplotype with or without SAVI-causing mutations resulted in significantly increased IFN-β1 expression in comparison with cells transfected with the H232 haplotype. Besides H232, the triple non-synonymous R71H, G230A and R293Q (HAQ) encoding allele is the second most occurring loss-of-function human *STING1* allele. Patel et al. showed that human HAQ/HAQ B cells have almost absent STING expression and no response to cyclic dinucleotides in terms of IFN-β production [E9]. Moreover, using a knock-in HAQ mouse, they recapitulated findings comparable to human HAQ/HAQ individuals [E9]. The loss-of-function property of the HAQ allele was confirmed in another study, where the HAQ allele was introduced into T-cells harboring the V155M STING mutant [6]. The HAQ polymorphism functionally inhibited STING signaling by the constitutive active V155M mutant, relocated it to the endoplasmic reticulum, restored T-cell proliferation and corrected NF-κB activation [6]. One SAVI patient with two *de novo* STING mutations (S102P and F279L) on the paternal chromosome and heterozygous in *trans* for the HAQ allele has been reported [E7]. The patient had lower basal IFN-β promotor activity and an infancy-onset phenotype. However, his overall clinical course was not mild as he suffered from ischemic stroke, severe cutaneous lesions including nasal septum perforation, respiratory and soft tissue infections and signs of pulmonary involvement. Since in our patient a relatively mild disease course without skin or pulmonary involvement and an adult-onset phenotype was observed, we tested for the presence of protective alleles. Surprisingly, WES revealed that he was homozygous for both IFIH1 risk alleles (encoding T946, R843; depth >30) and heterozygous for R232 encoding allele (depth >30) in STING despite his relatively mild phenotype. Given the limited reports on these disease modifying alleles in SAVI patients, the implications of these findings in terms of follow-up, treatment and screening examinations remain uncertain. As for now, the patient is followed up monthly and chest radiograph is performed yearly. Finally, environmental factors could influence the disease course of SAVI patients. Bennion et al. studied mice with heterozygous N153S mutations, corresponding with the N154S mutation in humans [E10]. Unlike SAVI patients, these mice did not develop pulmonary fibrosis in a specific-pathogen-free environment. However, when inoculating them intranasally with murine gammaherpesvirus 68 (γHV68) they developed pneumonia and pulmonary fibrosis in contrast to their wild type littermate controls. Furthermore, pulmonary fibrosis could be prevented by administration of cidofovir, an antiviral drug with activity against γHV68.

**Treatment**

Conventional anti-inflammatory treatments, including corticosteroids, disease-modifying anti-rheumatic drugs, anti-TNF, anti-CD20, or intravenous immunoglobulins had variable or limited effect on the disease course. Currently, treatment with JAK inhibitors is proposed, initially based on the observation that JAK inhibitors blocked *in vitro* *IFNB1* transcription in patients by 30-50% and the induction of IFN response genes by 60-90% [1]. Since then, several patients (n=20) have been treated with ruxolitinib (n=8), baricitinib (n=7), or tofacitinib (n=5).

Ruxolitinib is a JAK1/2 inhibitor that exerts potent anti-inflammatory and immunosuppressive effects [E12]. In 3 patients, ruxolitinib led to complete resolution of cutaneous lesions and improvement in lung function and general wellbeing allowing tapering and cessation of high-dose corticosteroids [E13], but in 5 others no effect was observed [E14], corticosteroids were required to treat cutaneous flares or relapse of ILD [4][E15] or even progressive vasculopathy occurred [E16]. However, in some patients ruxolitinib had to be lowered or stopped because of recurrent viral respiratory infections [E15], the need of routine immunizations [4], or were on suboptimal dosage [E16], possibly explaining the lack of efficacy.

Baricitinib is a structural analog of ruxolitinib and also functions as a JAK1/JAK2 inhibitor. Seven SAVI patients treated with baricitinib are reported in literature [E8, E2, E14, E17]. In two patients, baricitinib led to complete resolution of cutaneous symptoms [E8, E14]. One patient, who did not respond to ruxolitinib, became oxygen-free during baricitinib treatment and tapering of oral corticosteroids was possible [E14]. Sanchez et al. reported significant clinical improvement of cutaneous vasculitis in all SAVI patients [E17]. Flares still occurred, albeit with reduced duration and severity and further tissue loss was prevented. Three SAVI patients had baseline lung disease and in all of them improvement on baricitinib in terms of walking distance and pulmonary function was observed. In one of their patients who received corticosteroid treatment at baseline, corticosteroids could initially be tapered but prior to cessation, a dose escalation was necessary because of subjective respiratory difficulties. Despite clinical improvement, none of the SAVI patients in the trial met the remission criteria (defined as daily diary score <0.15, cessation of corticosteroids and CRP < 5mg/L). In another study, one patient with skin vasculopathy and alopecia was treated with baricitinib resulting in healing of a skin ulcer, hair regrowth and improved overall well-being [E2]. However, there was no visible change in the livedo, facial erythema, or laboratory parameters. Treatment with baricitinib in all studies was well tolerated. Skin, nail, and oral infections and upper respiratory tract infections were the most frequently reported adverse events [E17]. Viral reactivation with BK virus (BK viremia and viruria) was reported in 2 patients [E17] but no patients had to discontinue baricitinib treatment.

Different from ruxolitinib and baricitinib, tofacitinib has a selective action against JAK1 and JAK3 [E18]. In one patient, tofacitinib treatment improved the cutaneous lesions, but no respiratory improvement was noted [E7]. Tang et al. reported 2 patients on tofacitinib treatment [E19]. Both patients had radiologic progression of pulmonary lesions despite treament, and one patient developed progressive cutaneous lesions which required dose escalation of corticosteroids. One patient died because of respiratory failure and in the other patient tofacitinib was discontinued at the age of 74 months on the parents’ initiative. After reduction of prednisone and withdrawal of tofacitinib, the respiratory symptoms and arthritis did not deteriorate but the skin rashes progressed. König et al. reported two patients, predominantly suffering from distal ischemia, were tofacitinib had beneficial effects on pain relief, suggesting improved perfusion [E2]. No adverse events were reported, but the two affected individuals were only treated for a short duration of 17 days to assess the effect on the IFN signature.

More recently, progress in understanding the molecular mechanisms of SAVI and research on STING in malignancies [E20] might lead to more targeted therapeutic approaches. Highly potent and selective small-molecule antagonists of the STING protein have been developed [E21]. These small-molecules covalently target the predicted transmembrane cysteine residue 91 and block the activation-induced palmitoylation of STING, which is essential for its assembly into multimeric complexes at the Golgi apparatus and for the recruitment of downstream signaling factors. This results in a reduction of STING-mediated inflammatory cytokine production in both human and mouse cells. Furthermore, in *Trex1^−/−^* mice, STING inhibition resulted in a significant reduction in serum levels of type I IFNs and marked amelioration of systemic inflammation.

*Supplementary Table E1*

Summary of clinical data including treatment and technical investigations done in proband (III.1) and affected father (II.2).

|  | **III.1** | **II.2** |
| --- | --- | --- |
| **Clinical data** |  |  |
| Age of onset | Adult | Infancy |
| Genetics | p.V155M | p.V155M |
| Status | Alive | Died at 30 years due to respiratory failure |
| Skin | - | - |
| Pulmonary | - | + (interstitial lung disease, pulmonary fibrosis, diagnosis at 9y of age; hypoplastic left thorax, diagnosis in infancy) |
| Vascular | - | + (distal ischemia, amputation of lower extremities) |
| Musculoskeletal | - | - |
| Systemic | + (elevated CRP/ESR) | + (failure to thrive, febrile episodes, elevated CRP/ESR) |
| Renal | + (pauci-immune intra- and extracapillary glomerulonephritis) | + (pauci-immune intra- and extracapillary glomerulonephritis) |
| Auto-antibodies | + (p-ANCA, ANF) | + (c-ANCA, ANF) |
| Infections | - | + (SSTI, LRTI: pneumonia; RTI from early childhood onwards) |
| Other possible related features | + (iritis)  + (hemoptysis, 1 episode 11 months before admission) | + (pericarditis) |
| **Technical examinations** |  |  |
| Immunophenotype | CD4^+^ T-cell (159/µL, reference 455-1885/µL)  NK-cell (35/µL, reference 66-745/µL)  Tetanus toxoid stimulation (1.36 x 10^3^ cpm, reference ≥ 5)  PHA (95.30 x 10^3^ cpm, reference ≥ 5) | ND |
| Chest X-ray | Normal findings | Infiltrative opacification, mostly in the periphery |
| Chest high resolution computed tomography | Mild emphysema (contrast enhanced computed tomography).  Of note, patient reports active nicotine exposure. | Multiple hilar and mediastinal adenopathies, interstitial fibrosis |
| Pulmonary function test | Restrictive function (FVC 75%, FEV1 63%, TLC 73% of predicted values), normal diffusion per alveolar volume (TL_CO_ 55%, TL_CO_/AV 78% of predicted values) | Restrictive and obstructive lung function |
| Transthoracal echocardiography | ND | Concentric left ventricular hypertrophy, minimal mitral and tricuspid valve insufficiency |
| Electrocardiogram | ND | Left ventricular hypertrophy |
| Abdominal ultrasound | Normal spleen (craniocaudal diameter 13.5 cm, reference < 14 cm), mild hepatomegaly (18 cm midclavicular, reference < 16 cm) | Splenomegaly (craniocaudal diameter 15 cm, reference < 14 cm) and mild hepatomegaly (16 cm midclavicular, reference < 16 cm) |
| Gastroscopy | Normal findings | Reflux oesophagitis grade 1 |
| Colonoscopy | Normal findings | ND |
| Renal biopsy | Pauci-immune focal crescentic and necrotizing glomerulonephritis with mesangial C3 deposition without significant immunoglobulin depositions | Pauci-immune focal crescentic and necrotizing glomerulonephritis with mesangial C3 deposition and limited IgM deposition along the glomerular basal membrane |
| Lung biopsy | ND | Pulmonary hemosiderosis and interstitial fibrosis |
| **Treatment(s)** |  |  |
| Corticosteroids | + (500 mg pulse IV, followed by 70 mg prednisolone po tapered by 5 mg/week) | + (600 mg pulse IV 1 week at 10 years, followed by prednisone 40 mg po tapered to 2.5 mg) |
| Anti-CD20 | + (375 mg/m² every week for 4 weeks) | - |
| Azathioprin | + (2 mg/kg) | + (50 mg once daily) |
| Cyclosporin | NA | + (dosage based on therapeutic monitoring) |
| Plasmapheresis | NA | + |
| JAK inhibitors | - (given the SAVI diagnosis after treatment and good clinical response with conventional treatment, JAK inhibition was not evaluated). | NA |
| **Treatment response** |  |  |
| Renal | Resolution of hematuria and pyuria, proteinuria decreased to 0.457 g/L creatinine (baseline 1.09 g/L creatinine) at 16 months follow up | End stage kidney disease, requiring dialysis (at age of 11 years) and kidney transplantation (at age of 12 years), returned to dialysis (at age of 25 years) due to graft rejection |
| Pulmonary | NA | Progressive disease during adolescence and adulthood, worsening of pulmonary function tests |
| Vascular | NA | Progressive disease, recurrent thrombosis of arteriovenous fistula, lower limb ischemia requiring amputation at 29 years of age. |

*Abbreviations*: ESR: erythrocyte sedimentation rate, ND: not determined, NA: not applicable, po: per os, SSTI: skin and soft tissue infection, LRTI: lower respiratory tract infection, FVC: forced vital capacity, FEV1: forced expiratory volume at 1 sec, TLC: total lung capacity, TL_CO_: carbon monoxide transfer capacity; AV: alveolar volume .

*Supplementary Figure E1*

**Graphical visualization of clinical and genetic characteristics of SAVI patients (n=56).**


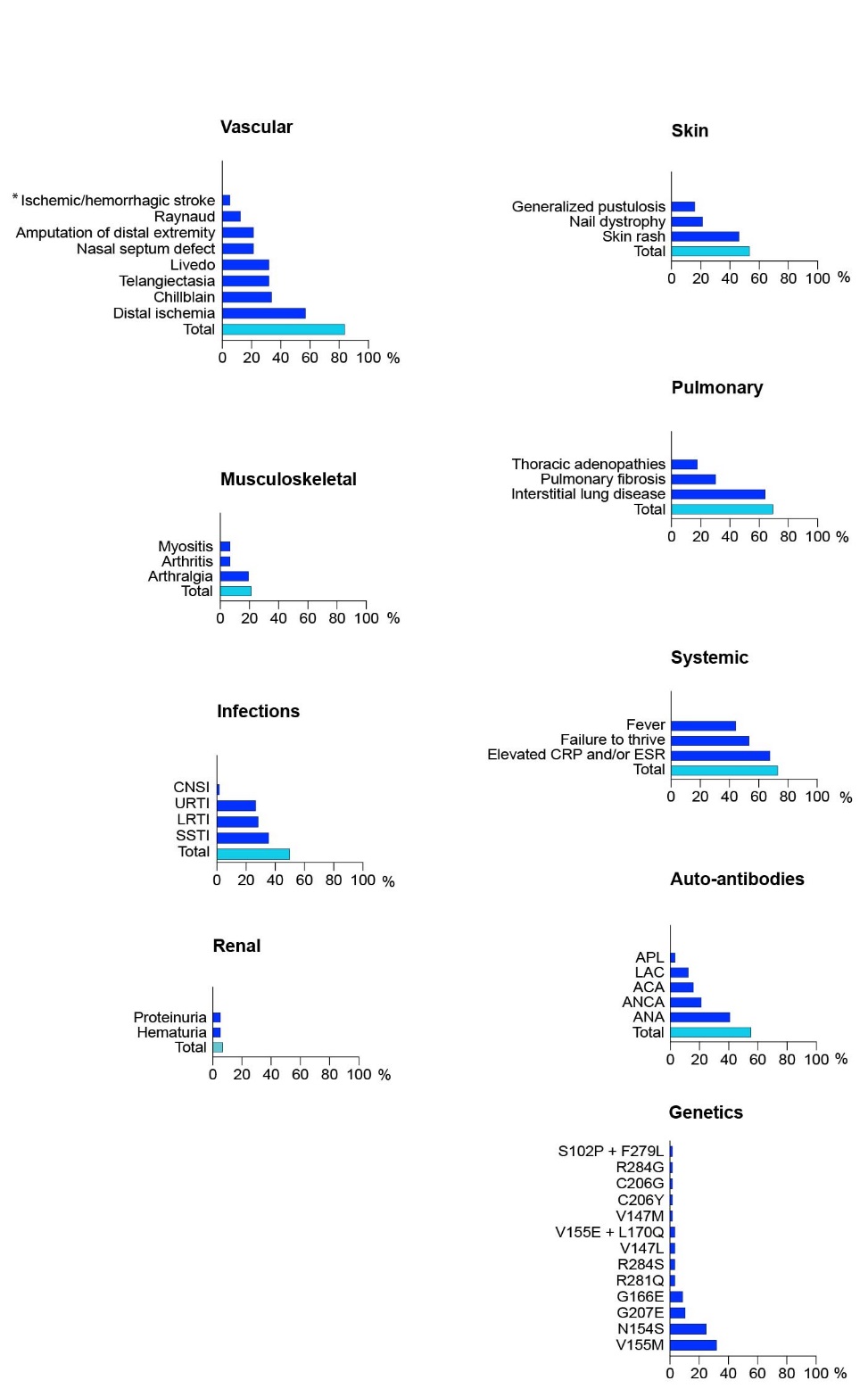


*Abbreviations*: CRP: C-reactive protein, ESR: erythrocyte sedimentation rate, ANA: antinuclear antibody, ANCA: antineutrophil cytoplasmic antibody, APL: antiphospholipid antibody, LAC: lupus anticoagulant, ACA: anti cardiolipin antibody, CNS: central nervous system, SSTI: skin and soft tissue infections, LRTI: lower respiratory tract infections, URTI: upper respiratory tract infections. * One case, not included under ischemic stroke, reports suggestive ischemic lesions on brain MRI but this was not confirmed by angio-MRI [4]. Total (from n=56) reports the percentage of patients with at least one feature within the respective manifestation.

**References**

E1. Li, Y., H.L. Wilson, and E. Kiss-Toth, *Regulating STING in health and disease.* Journal of Inflammation, 2017. **14**(1): p. 11.

E2. König, N., et al., *Familial chilblain lupus due to a gain-of-function mutation in STING.* Annals of the Rheumatic Diseases, 2017. **76**(2): p. 468.

E3. Keskitalo, S., et al., *Novel TMEM173 Mutation and the Role of Disease Modifying Alleles.* Frontiers in Immunology, 2019. **10**: p. 2770.

E4. Melki, I., et al., *Disease-associated mutations identify a novel region in human STING necessary for the control of type I interferon signaling.* Journal of Allergy and Clinical Immunology, 2017. **140**(2): p. 543-552.e5

E5. Zampeli, E., et al., *Abstract 07.13 A case of sting-associated vasculopathy with onset in infancy (savi) in a young adult male with a novel tmem173 gene mutation*. Ann Rheum Dis, 2017. **76**(Suppl 1): A1-A103.

E6. Konno, H., et al., *Pro-inflammation Associated with a Gain-of-Function Mutation (R284S) in the Innate Immune Sensor STING.* Cell Reports, 2018. **23**(4): p. 1112-1123.

E7. Seo, J., et al., *Tofacitinib relieves symptoms of stimulator of interferon genes (STING) associated vasculopathy with onset in infancy caused by 2 de novo variants in TMEM173.* Journal of Allergy and Clinical Immunology, 2017. **139**(4): p. 1396-1399.e12.

E8. Chia, J., et al., *Failure to thrive, interstitial lung disease, and progressive digital necrosis with onset in infancy.* Journal of the American Academy of Dermatology, 2016. **74**(1): p. 186-189.

E9. Patel, S., et al., *The Common R71H-G230A-R293Q Human TMEM173 Is a Null Allele.* The Journal of Immunology, 2017. **198**(2): p. 776.

E10. Bennion, B.G., et al., *A Human Gain-of-Function STING Mutation Causes Immunodeficiency and Gammaherpesvirus-Induced Pulmonary Fibrosis in Mice.* J Virol, 2019. **93**(4).

E11. Malle, L., et al., *Interstitial lung disease in STING-associated vasculopathy with onset in infancy (SAVI): preliminary genotype-phenotype correlation.* Pediatric Rheumatology Online Journal, 2015. **13**(Suppl 1): p. O32-O32.

E12. Elli, E.M., et al., *Mechanisms Underlying the Anti-inflammatory and Immunosuppressive Activity of Ruxolitinib.* Frontiers in Oncology, 2019. **9**: p. 1186.

E13. Fremond, M.L., et al., *Efficacy of the Janus kinase 1/2 inhibitor ruxolitinib in the treatment of vasculopathy associated with TMEM173-activating mutations in 3 children.* J Allergy Clin Immunol, 2016. **138**(6): p. 1752-1755.

E14. Balci, S., et al., *Baricitinib experience on STING-associated vasculopathy with onset in infancy: A representative case from Turkey.* Clinical Immunology, 2020. **212**: p. 108273.

E15. Volpi, S., et al., *Efficacy and Adverse Events During Janus Kinase Inhibitor Treatment of SAVI Syndrome.* J Clin Immunol, 2019. **39**(5): p. 476-485.

E16. Saldanha, R.G., et al., *A Mutation Outside the Dimerization Domain Causing Atypical STING-Associated Vasculopathy With Onset in Infancy.* Front Immunol, 2018. **9**: p. 1535.

E17. Sanchez, G.A.M., et al., *JAK1/2 inhibition with baricitinib in the treatment of autoinflammatory interferonopathies.* The Journal of Clinical Investigation, 2018. **128**(7): p. 3041-3052.

E18. Hodge, J.A., et al., *The mechanism of action of tofacitinib - an oral Janus kinase inhibitor for the treatment of rheumatoid arthritis.* Clin Exp Rheumatol, 2016. **34**(2): p. 318-28.

E19. Tang, X., et al., *STING-Associated Vasculopathy with Onset in Infancy in Three Children with New Clinical Aspect and Unsatisfactory Therapeutic Responses to Tofacitinib.* J Clin Immunol, 2019

E20. Yang, H., et al., *STING activation reprograms tumor vasculatures and synergizes with VEGFR2 blockade.* The Journal of Clinical Investigation, 2019. **129**(10): p. 4350-4364.

E21. Haag, S.M., et al., *Targeting STING with covalent small-molecule inhibitors.* Nature, 2018. **559**(7713): p. 269-273.
